# Transcriptomic, proteomic and ultrastructural studies on salinity-tolerant *Aedes aegypti* in the context of rising sea levels and arboviral disease epidemiology

**DOI:** 10.1186/s12864-021-07564-8

**Published:** 2021-04-09

**Authors:** Ranjan Ramasamy, Vaikunthavasan Thiruchenthooran, Tibutius T. P. Jayadas, Thampoe Eswaramohan, Sharanga Santhirasegaram, Kokila Sivabalakrishnan, Arunasalam Naguleswaran, Marilyne Uzest, Bastien Cayrol, Sebastien N. Voisin, Philippe Bulet, Sinnathamby N. Surendran

**Affiliations:** 1 ID-FISH Technology Inc., Milpitas, CA 95035 USA; 2 Department of Zoology, University of Jaffna, Jaffna, Sri Lanka; 3 Institute of Cell Biology, University of Bern, Baltzerstrasse 4, CH-3012 Bern, Switzerland; 4 UMR BGPI, University of Montpellier, INRAE, CIRAD, SupAgro, Montpellier, France; 5 Platform BioPark Archamps, Archamps, France; 6 CR Université Grenoble Alpes, Institute for Advanced Biosciences, Inserm U1209, CNRS UMR 5309, Grenoble, France

**Keywords:** *Aedes aegypti*, Arboviral diseases, Climate change, Coastal salinity, Cuticle proteomics, Cuticle ultrastructure, Insecticide resistance, Rising sea levels, Transcriptomics, Salinity tolerance

## Abstract

**Background:**

*Aedes aegypti* mosquito, the principal global vector of arboviral diseases, lays eggs and undergoes larval and pupal development to become adult mosquitoes in fresh water (FW). It has recently been observed to develop in coastal brackish water (BW) habitats of up to 50% sea water, and such salinity tolerance shown to be an inheritable trait. Genomics of salinity tolerance in *Ae. aegypti* has not been previously studied, but it is of fundamental biological interest and important for controlling arboviral diseases in the context of rising sea levels increasing coastal ground water salinity.

**Results:**

BW- and FW-*Ae. aegypti* were compared by RNA-seq analysis on the gut, anal papillae and rest of the carcass in fourth instar larvae (L4), proteomics of cuticles shed when L4 metamorphose into pupae, and transmission electron microscopy of cuticles in L4 and adults. Genes for specific cuticle proteins, signalling proteins, moulting hormone-related proteins, membrane transporters, enzymes involved in cuticle metabolism, and cytochrome P450 showed different mRNA levels in BW and FW L4 tissues. The salinity-tolerant *Ae. aegypti* were also characterized by altered L4 cuticle proteomics and changes in cuticle ultrastructure of L4 and adults.

**Conclusions:**

The findings provide new information on molecular and ultrastructural changes associated with salinity adaptation in FW mosquitoes. Changes in cuticles of larvae and adults of salinity-tolerant *Ae. aegypti* are expected to reduce the efficacy of insecticides used for controlling arboviral diseases. Expansion of coastal BW habitats and their neglect for control measures facilitates the spread of salinity-tolerant *Ae. aegypti* and genes for salinity tolerance. The transmission of arboviral diseases can therefore be amplified in multiple ways by salinity-tolerant *Ae. aegypti* and requires appropriate mitigating measures. The findings in *Ae. aegypt*i have attendant implications for the development of salinity tolerance in other fresh water mosquito vectors and the diseases they transmit.

**Supplementary Information:**

The online version contains supplementary material available at 10.1186/s12864-021-07564-8.

## Background

From an origin in tropical forests where it blood fed on animals, *Aedes aegypti* adopted a preference for developing near human habitations and blood feeding on humans, and spread widely to become the principal vector of important arboviral diseases including dengue, chikungunya, yellow fever, and Zika [1–3]. It is regarded as an obligate fresh water (FW) mosquito that lays eggs (oviposits) and undergoes larval and pupal (preimaginal) development in natural (e.g. rainwater pools, leaf axils) and anthropogenic (e.g. water storage tanks, discarded containers) FW collections near human habitation [4–8]. Larval source reduction efforts, critically important for controlling arboviral diseases, presently only target such FW habitats of *Ae. aegypti* and the secondary arboviral vector *Aedes albopictus* [6–8]. The two *Aedes* vectors were recently shown to oviposit and undergo preimaginal development in coastal anthropogenic brackish water (BW) habitats (e.g. beach litter, coastal wells) in the Jaffna peninsula of Sri Lanka [9–11], with fresh, brackish and saline water defined as containing < 0.5 ppt (parts per thousand), 0.5-30 ppt and > 30 ppt salt, respectively [9]. Development of the *Aedes* vectors in coastal BW has since been observed in Brunei [12], USA [13], Brazil [14] and Mexico [15].

*Aedes aegypti* oviposits in up to 18 ppt salt and shows 100% survival of first instar larvae to adulthood in 12 ppt salt and partial survival in 20 ppt salt in the Jaffna peninsula [9–11]. Preimaginal stages of BW *Ae. aegypti* have an inheritable higher LC_50_ for salinity than FW *Ae. aegypti* [16]. Colonies of salinity-tolerant *Ae. aegypti* tend to prefer BW to FW for oviposition [16], develop larger anal papillae [17] and can be infected with dengue virus [18]. Development of *Ae. aegypti* and *Ae. albopictus* in BW increases the potential for arboviral disease transmission which can be exacerbated by rising sea levels due to global warming causing greater salinization of inland waters [19–23]. The 1130km^2^ Jaffna peninsula in northern Sri Lanka is undergoing rapid salinization of its groundwater aquifers and coastal wells due to the incursion of sea water [20, 24]. Genetic changes for salinity tolerance can therefore rapidly spread among *Ae. aegypti* populations within this small peninsula, increasing the transmission and prevalence of dengue and chikungunya that are endemic in the peninsula [9, 18, 24].

Most mosquito species oviposit and undergo preimaginal development to adulthood in FW but about 5% develop in brackish or saline water [25]. Some salinity-tolerant species are vectors of important human diseases e.g. *Anopheles merus*, *Anopheles albimanus* and *Anopheles sundaicus* malaria vectors in Africa, the Americas and Asia respectively [19, 20, 22]. The major Asian malaria vectors *Anopheles culicifacies* and *Anopheles stephensi*, considered obligate FW mosquitoes like *Ae. aegypti*, have also recently been observed to develop in coastal BW in the Jaffna peninsula [11, 26–28].

All mosquito larvae need to osmoregulate to maintain haemolymph composition and osmolarity [29]. Water enters *Ae. aegypti* larvae in FW by diffusion through the cuticle and during feeding, while ions are lost by diffusion. Larvae in FW therefore produce a dilute urine and accumulate ions by active transport. *Aedes aegypti* larval structures regulating water and ion exchange with the environment are the midgut, Malpighian tubules, rectum, anal papillae and gastric caeca [29, 30]. The rectum of FW culicine mosquitoes like *Ae. aegypti* is structurally uniform and absorbs Na^+^ and Cl^−^ from urine produced by Malpighian tubules [29, 31]. The anal papillae also actively absorb Na^+^ and Cl^−^ from the surrounding FW [32–34]. Typical BW culicine mosquitoes (e.g. *Aedes tarsalis*) and BW anopheline mosquitoes (e.g. *An. albimanus*) possess specialized recta excreting a hypertonic, salt-rich urine for osmoregulation [29, 31]. Fourth instar larvae (L4) of FW *Ae. aegypti* are able to maintain haemolymph osmolarity (~ 300 mOsm equivalent to ~ 10 ppt salt or ~ 30% sea water) [29] for a short period by increasing amino acid and ion concentrations up to an external salinity of ~ 30% sea water [35–37]. Genomic changes and physiological mechanisms that permit FW *Ae. aegypti* and FW anopheline malaria vectors to oviposit and develop into adults in field habitats of up to 15 ppt salt (i.e. ~ 50% sea water) [9–16, 26–28] are however not known. We therefore compared in long-term BW- and FW-adapted *Ae. aegypti* (i) the mRNA levels in three L4 larval structures viz. the whole gut including associated Malpighian tubules (termed gut), anal papillae, and the rest of the carcass (termed carcass) using high-throughput RNA-seq, (ii) the proteomes of the cuticles shed when L4 become pupae, and (iii) the cuticles of L4 larvae and adult females by transmission electron microscopy (TEM). The findings from these studies are reported here in the context of the biology of salinity tolerance in *Ae. aegypti* and transmission of arboviral diseases.

## Results

### Transcripts for some cuticle proteins, notably RR-2s, are greatly increased in the L4 of salinity-tolerant *Ae. aegypti*

RNA-seq analysis resulted in 30,485 transcripts being mapped in the gut, anal papilla and carcass of *Ae. aegypti* L4 (Additional file S1). Differentially-spliced transcripts from the same gene were expressed with similar reads per million mapped reads (rpms) in any one structure with few exceptions. Transcript rpms from a gene varied between the three structures and sometimes between BW and FW L4. The ratio of rpms in BW to FW L4 termed fold change (FC) were calculated for every transcript (Additional file S1). All transcripts with highly increased (FC > 100) or decreased (FC ≤ 0.01) levels in L4 of BW *Ae. aegypti*, and the detection of corresponding proteins in shed L4 cuticles by proteomics, are listed in Additional file S2. Transcripts, including multiple transcripts from the same gene, for several cuticle proteins were increased in BW with FC > 100 in all three structures and these are summarized in Table 1. *Aedes aegypti* cuticle proteins shown in Table 1 were classified into families by homology with * Anopheles gambiae* cuticle protein families [38, 39], viz. RR-1 and RR-2 containing two forms of the Rebers and Riddiford consensus sequence [40] comprising ≥156 cuticle proteins in *An. gambiae*; CPF containing a highly conserved region of ~ 44 amino acids; CPFL (CPF-like in a conserved C-terminal region); TWDL (Tweedle) from a characteristic *Drosophila* mutant; five families in addition to TWDL with significant low complexity sequences, viz. CPLCA, CPLCG, CPLCW, CPLCP rich in alanine, glycine, tryptophan and proline respectively, and an unclassified family CPLCX; two families of cuticle proteins analogous to peritrophins CPAP1 and CPAP3 with one and three chitin-binding domains respectively; and CPCFC containing 2 or 3 C-x(5)-C repeats. Chitin-binding properties are ascribed to RR-1, RR-2, CPAPs, CPCFC, CPFL and TWDL families [39]. Some mosquito cuticle proteins remain unclassified [38, 39] and are termed CPX. Resilin, elastin and cuticulin are proteins that have structural roles in the cuticle [38–41], while others like dumpy [39], Osiris proteins [42], cytoskeleton and muscle proteins, golgin, extensin, C-type lectin, protein target of myb-membrane trafficking, oxygenases, adhesins, oxidases, fatty acid synthase, long chain fatty acid elongase, glucose dehydrogenase and proteases function in cuticle formation, or its digestion during ecdysis, and are variably detected in cuticle preparations [38, 39]. These are collectively termed as other proteins associated with cuticles or OPACs. Pertinent OPACs with marked FC changes are discussed in a separate section below.

**Table 1:** Cuticle Protein Genes with Transcripts showing FC > 100 in BW *Ae. aegypti* L4

Gene Category	Carcass		Anal Papilla		Gut	
	No. of Genes	No. of Transcripts	No. of Genes	No. of Transcripts	No. of Genes	No. of Transcripts
**All genes**	63	70	51	61	48	54
**RR-1 family**	1	1	0	0	2	2
**RR-2 family**	22	25	15	15	8	8
**CPLCP family**	0	0	1	1	8	8
**TWDL family**	0	0	2	3	3	5
**CPAPs**	0	0	0	0	1	1
**CPX**	8	8	0	0	0	0

Table 1 shows that many genes coding for cuticle proteins, particularly members of the RR-2 family, were among the genes with transcripts showing FC > 100*.* Transcripts for cuticle proteins formed a significant proportion of all transcripts with FC > 100 in carcass (49%), anal papilla (31%) and gut (44%). Transcripts for RR2s formed a large majority of the cuticle protein transcripts with FC > 100 in carcass (74%) and anal papilla (79%). Transcripts for RR-2s and CPLCPs constituted 33% each of all cuticle protein transcripts with FC > 100 in gut. Fewer transcripts were strongly decreased with FC ≤ 0.01 in the three structures, including mRNAs for two serine/threonine protein kinases in carcass, nine serine/threonine protein kinases in gut, an RR2 each in carcass and anal papilla, and two GTP-coupled signaling proteins in gut (Additional file S2). Some cuticle protein transcripts with FC > 100 or ≤ 0.01 in either anal papilla, carcass or gut, had different expression levels in the three structures, with extreme differences in transcripts for four RR-2s and one RR-1 that had FC > 100 in carcass and ≤ 0.1 in gut (highlighted in Additional File S2). Transcripts for two RR-2s had FC > 100 in all three structures. Transcripts for 11 other RR-2s, two TWDLs, two CPLCPs, as well as a cuticulin and a resilin classified as OPACs, had FC > 100 in two of three structures (Additional file S2).

Some of the large changes of FC > 100 for cuticle protein transcripts reported in Table 1 arise from transcripts expressed at low rpms in FW (Additional file S2). We reasoned that cuticle protein transcripts with the highest abundances measured as rpm may reflect important cuticle functions, and therefore analyzed the ten most abundant cuticle protein transcripts in each of the three structures in both BW and FW L4. The results of this analysis presented in Table 2 identified some transcripts that were not among those with FCs > 100 listed in Additional file S2 and summarized in Table 1. All cuticle protein genes in Table 2 only showed a single transcript in the RNA-seq analysis. Some transcripts with top ten rpms in the three structures in FW are expressed with FC < 1, likely reflecting a relative down regulation in expression of the corresponding genes in BW. There was also a marked shift towards more RR-2 transcripts accompanied by large FCs in the top ten transcripts in BW L4 when compared with the top ten transcripts in FW L4. This was particularly striking for anal papilla where among the top ten abundant transcripts, there were seven RR-1 and three CPLCG transcripts in FW L4, compared with six RR-2 and four RR-1 transcripts in BW L4. Some top ten expressed transcripts in BW were structure-specific e.g. an AAEL009001 transcript for a RR-2 increased in expression only in gut, or structure-shared e.g. an AAEL004746 transcript for a RR-2 increased in expression in all three structures. Cuticle proteins encoded by most of the top ten abundant transcripts in all three structures in FW were detected by proteomics in shed L4 cuticles (proteomics data are presented in a separate section below). The transcript for the 40S ribosome S7 gene AAEL009496, considered as an internal control, was expressed at similar abundances in each of the three structures in BW and FW L4 with FCs of 0.6 to 0.7.

**Table 2:** Top Ten Cuticle Protein Transcripts by RPM in Carcass, Anal Papilla and Gut

**Carcass TOP 10 BW**				**Carcass TOP 10 FW**			
**rpm**	**FC**	**Gene**	**Cuticle protein family**	**rpm**	**FC**	**Gene**	**Cuticle protein family**
1626	0.6	Ribosomal S7	na	2561	0.6	Ribosomal S7	na
1725	143	AAEL015163^a^	RR-2	1592	0.6	AAEL013512^a^	RR-1
1626	351	AAEL009784^a^	RR-2	1564	0.4	AAEL013520^a^	RR-1
1522	708	AAEL009801^a^	RR-2	1110	0.6	AAEL003239^b^	RR-1
996	28	AAEL003049^b^	RR-1	721	0.9	AAEL011444^a^	RR-1
963	19	AAEL009793^a^	RR-2	221	0.3	AAEL013517^a^	RR-1
958	186	AAEL004780^a^	RR-2	67	0.9	AAEL002110^b^	RR-2
931	0.6	AAEL013512^a^	RR-1	63	5	AAEL008289^a^	RR-1
700	593	AAEL004746	RR-2	56	0.1	AAEL009796^a^	RR-2
659	0.9	AAEL011444^a^	RR-1	50	19	AAEL009793^a^	RR-2
614	0.6	AAEL003239^b^	RR-1	43	1	AAEL002231	CPLCG
**Anal Papilla TOP 10 BW**				**Anal Papilla TOP 10 FW**			
**rpm**	**FC**	**Gene**	**Cuticle protein family**	**rpm**	**FC**	**Gene**	**Cuticle protein family**
1629	0.6	Ribosomal S7	na	2642	0.6	Ribosomal S7	na
1062	0.7	AAEL013512^a^	RR-1	1594	0.7	AAEL013512^a^	RR-1
1023	37	AAEL011504^a^	RR-2	1585	0.6	AAEL011444^a^	RR-1
921	0.6	AAEL011444^a^	RR-1	1553	0.6	AAEL013520^a^	RR-1
879	0.6	AAEL013520^a^	RR-1	1183	0.7	AAEL003239^b^	RR-1
793	0.7	AAEL003239^b^	RR-1	1095	0.1	AAEL003242^a^	RR-1
635	269	AAEL004746	RR-2	460	0.1	AAEL002211^b^	CPLCG
543	1392	AAEL004770	RR-2	334	0.5	AAEL003049^b^	RR-1
431	141	AAEL004745	RR-2	250	0.1	AAEL002229	CPLCG
203	520	AAEL004772	RR-2	198	0.04	AAEL002191^a^	CPLCG
193	140	AAEL004751	RR-2	145	0.3	AAEL013517^a^	RR-1
**Gut top 10 BW**				**Gut top 10 FW**			
**rpm**	**FC**	**Gene**	**Cuticle protein family**	**rpm**	**FC**	**Gene**	**Cuticle protein family**
1911	0.7	Ribosomal S7	na	2657	0.7	Ribosomal S7	na
1230	1.3	AAEL013512^a^	RR-1	1462	0.4	AAEL013520^a^	RR-1
802	1.5	AAEL003239^b^	RR-1	919	1.3	AAEL013512^a^	RR-1
575	0.4	AAEL013520^a^	RR-1	555	1.5	AAEL003239^b^	RR-1
443	1.1	AAEL011444^a^	RR-1	400	1.1	AAEL011444^a^	RR-1
212	171	AAEL004770	RR-2	49	0.3	AAEL013517^a^	RR-1
204	44	AAEL004746	RR-2	34	0.1	AAEL003242^a^	RR-1
173	199	AAEL009001^b^	RR-2	28	0.2	AAEL015163^a^	RR-2
135	52	AAEL004745	RR-2	25	0.1	AAEL009801^a^	RR-2
68	93	AAEL000085	CPX	14	0.7	AAEL007194^a^	RR-1
66	11	AAEL011504^a^	RR-2	14	0.5	AAEL009784^a^	RR-2

Legend to Table 2: *rpm* reads per million mapped reads, *FC* fold change in rpm in BW compared to FW, *na* not applicable, S7 is the cytoplasmic 40S ribosomal protein coded for by its single transcript AAEL009496-RA; ^a^ detected by proteomic analysis in both shed L4 BW and FW cuticles; ^b^detected by proteomic analysis only in shed L4 BW cuticles

### Shed BW and FW L4 cuticles are different by proteomics analysis

There were 607 unique proteins consistently identified in all three technical replicates of a biological replicate in both BW and FW shed L4 cuticles by proteomics (Additional file S3). Of these, 266 were detected only in BW cuticles and 23 only in FW cuticles. Among the 607 proteins, there were 103 cuticle proteins of which 21 were detected only in BW cuticles and none only in FW cuticles. Amongst the 103 cuticle proteins, the more numerous were 33 RR-1s, 32 RR-2s, ten CPLCGs, nine CPAPs, and seven CPCLWs (Additional file S3). The 21 BW cuticle-specific cuticle proteins were composed of 10 RR-1s, seven RR-2s, three CPLCGs and one CPAP1. Many OPACs were amongst the 504 proteins other than cuticle proteins uniquely identified in cuticles (data in ProteomeXchange repository).

### BW-specific cuticle proteins identified by proteomics in shed L4 cuticles and their relative transcript levels in L4

Of the 21 cuticle proteins specifically identified only in BW L4 cuticles, a CPLCG and two RR-1s had transcript levels with FC < 1 in all three structures (Additional file S3). Of the 21 BW-specific cuticle proteins identified by proteomics that had transcripts with FC > 10 in any structure, five were in carcass (two of these concomitantly in gut), one in anal papilla and three in gut. Transcript levels for nine of the 21 BW cuticle-specific cuticle proteins showed prominent differences between the three structures as exemplified by AAEL003272 coding for a RR-1 with FC 777 in carcass that had corresponding FCs < 1 in anal papilla and gut (Additional file S3).

### Transcriptomic analysis shows differences in mRNA levels for pertinent non-cuticle proteins and long non-coding RNAs in BW and FW L4

Transcriptomics and proteomics data for selected OPACs and proteins other than cuticle proteins with potential roles in salinity adaptation as well as transcriptomic data for long non-coding RNA are summarized below.

#### Long non-coding RNAs

Several long non-coding RNAs (lncRNAs) that may regulate gene expression at the chromosome, transcription and post-transcription levels, were highly increased (FC > 100) or highly decreased (FC ≤ 0.01) in different structures (Additional file S2). Some lncRNAs with such large FC changes showed marked variations in FCs between the three structures (highlighted in Additional file S2). Many other lncRNAs had intermediate FC changes, and some of these also showed considerable variation in FCs between structures (Additional file S1).

#### Membrane receptors

Transcripts for a notch homologue receptor (FC > 100 anal papilla and carcass; FC 40 gut) and a frizzled transmembrane receptor (FC > 100 carcass; FC 31 gut; FC 32 anal papilla) were prominently increased in all three L4 structures, while transcripts for two G-protein-coupled receptors and a putative odorant binding protein were strongly decreased in gut (FC 0.01) and with FC < 1 in anal papilla and carcass (Additional file S2). Transcripts for a ppk301 sodium channel protein with a salinity-sensing role in oviposition [43] were expressed with FC 1 and very low rpm of 0.1 in all three structures (Additional file S4). None of these proteins were detected in shed L4 cuticles (data in ProteomeXchange repository).

#### Transcription regulatory proteins

Transcripts for a zinc finger and a bHLH transcription factors, CREB regulatory factor, speckle-type transcription regulator, a putative RNA-binding protein, and a different transcriptional regulator were markedly increased in all three structures (Additional file S2). A POU-domain containing transcription factor class 3 transcript was increased modestly in all three structures (Additional file S4). These proteins were not detected in shed cuticles (data in ProteomeXchange repository).

#### Signalling pathway proteins

Transcripts for a rho guanine nucleotide exchange factor in carcass, a cell polarity regulator protein par-6, a N-myc downstream regulator and a target of myb1 in membrane trafficking in anal papilla were greatly increased with FC > 100 (Additional file S2). Nine different serine/threonine protein kinases in gut and two others in carcass were strongly decreased (FC ≤ 0.01). These proteins were not detected in shed cuticles (data in ProteomeXchange repository).

Transcripts coding for MAP3K interacting protein, tak1-binding protein, MAP2K, Jun kinase, Jun, Kras GTPase and Rho GTPase were implicated in a short-term salinity response in anopheline L4 [44]. These seven proteins were not detected in shed L4 cuticles (data in ProteomeXchange repository), and their transcripts in BW L4 were either unchanged or modestly increased in the case of MAP2K, Jun kinase, Jun*,* and Kras GTPase with more marked increases in Rho GTPase (Additional file S4).

#### Moulting-related hormones and associated proteins

Data in Additional file S4 show that transcripts from three genes annotated as coding for eclosion hormones were expressed at low levels and either decreased or unchanged in BW L4. The transcript for the ecdysis-triggering hormone was increased in all three structures in BW L4. Transcripts from three genes annotated as coding for proteins induced by the moulting hormone ecdysone were markedly increased in BW L4 in all three structures. Changes in transcripts for 24 genes annotated as coding for proteins regulated by or binding the juvenile hormone (JH) were variably altered in BW L4, e.g. transcripts for a JH-regulated serine protease (FC 0.1–0.2) and JH acid methyl transferase (FC 0.2–0.4) were decreased in all three structures (Additional file S4), while transcripts for a haemolymph JH-binding protein was highly increased in carcass (FC 115) and also increased in gut and anal papilla (Additional file S2). Transcripts for a high affinity nuclear JH-binding protein were increased in all three structures in BW L4. None of these proteins were detected in shed L4 cuticles (data in ProteomeXchange repository).

#### Cytochrome P450

Transcripts from 135 cytochrome P450 genes were identified in the RNA-seq analysis (Additional file S1). Transcripts from two cytochrome P450 genes annotated as CYP18A1 in *Ae. aegypti* (FCs 11–111) and homologue of CYP4G17 in *An. gambiae* (FCs 14–71) were markedly increased in all three structures in BW L4 (Additional file S4). They were not found in shed L4 cuticles (data in ProteomeXchange repository).

#### Aquaporins (AQPs)

Transcripts for AQP3 and a putative AQP (AAEL021132) with FCs of 5–12 and 4–7 respectively, were increased in all three structures in BW L4 (Additional file S4). AQP1 and AQP4 transcripts were increased in anal papilla and carcass with FCs < 3. AQP6 transcript was decreased in anal papilla (FC 0.3). Only a single aquaporin, AQP2, was detected in both BW and FW shed L4 cuticles (data in ProteomeXchange repository).

#### V-type H^+^ transporter

Among its many components, only the proteolipid and catalytic subunit A were detected in BW and FW shed L4 cuticles (data in ProteomeXchange repository). Although transcripts were expressed at very high levels (e.g. rpm of 4138 in BW anal papilla for the proteolipid subunit), the FCs were 1–2 in BW L4 (Additional file S4).

#### Na^+^/K^+^ ATPase

Only the α and β2 subunits were detected in both BW and FW shed L4 cuticles (data in ProteomeXchange repository). Multiple transcripts for α were increased in all three structures with FCs up to 7, 6 and 12 in gut, anal papilla and carcass respectively while the single transcript for β2 had FCs of 3, 2 and 1 in gut, anal papilla and carcass respectively (Additional file S4).

#### Anion exchange protein

The protein had multiple transcripts. The majority of transcripts were either unchanged or modestly increased in the three structures in BW. One transcript RL was markedly increased in all three structures, and another RK was relatively prominently increased in anal papilla in BW (Additional file S4). The protein was not detected in shed L4 cuticles (data in ProteomeXchange repository).

#### Na^+^/H^+^ antiporters

NHE1, NHE2 and NHE3 proteins were not detected in shed L4 cuticles (data in ProteomeXchange repository). Many transcripts for NHE1 and NHE2 were expressed with relatively unchanged FCs in all structures in BW L4. The numerous transcripts of NHE3 were expressed with relatively low rpms but increased up to FC7, 5 and 12 in gut, anal papilla and carcass respectively, except for transcript RC which was markedly increased in gut (FC 44), anal papilla (FC 45) and carcass (FC 34) in BW L4 (Additional file S4).

#### NH_4_^+^ and amino acid transporters

The four NH_4_^+^ transporters AeAmt1, AeAmt2, AeRh50.1 and AeRh50.2 were not detected in shed L4 cuticles (data in ProteomeXchange repository). Their transcripts were relatively unchanged, except for AeRh50.2 which was markedly reduced (FC 0.1–0.4), in all three structures in BW L4 (Additional file S4). Transcript for a cationic amino acid transporter was however highly increased in anal papilla (FC 142) and also increased in gut (FC 8) and carcass (FC 21) in BW L4 (Additional file S2) but the protein was not identified in shed L4 cuticles (data in ProteomeXchange repository).

#### Allantoinase

Although transcripts were increased in all three structures in BW L4 (FC 3–6) as shown in Additional file S4, the protein was not detected in shed L4 cuticles (data in ProteomeXchange repository).

#### Chitin synthase

Seven transcripts identified were expressed with modest rpms but consistently increased in all three structures in BW L4, particularly transcript RD in gut (FC 23), anal papilla (FC 4) and carcass (FC 5) as shown in Additional file S4. The protein was not detected in shed L4 cuticles (data in ProteomeXchange repository).

#### Chitinase

Transcripts for chitinase were increased only in anal papilla (FC 3) in BW L4 (Additional file S4). The protein was detected in both BW and FW shed cuticles (data in ProteomeXchange repository).

#### Chitin-binding proteins

The transcript from AAEL012648 annotated as coding for a chitin-binding protein was markedly increased in gut (FC 188) and increased in anal papilla (FC 4) and carcass (FC 2) in BW L4 (Additional file S2). The protein was only detected in BW shed L4 cuticle (data in ProteomeXchange repository).

#### Other enzymes

Two transcripts for a very long chain fatty acid elongase (AAEL024147) were markedly increased in BW L4 in anal papilla (FC 145,126), gut (FC 33, 26) and carcass (FC 37, 28); for a fatty acid synthase (AAEL002228) in carcass (FC 113), gut (FC10) and anal papilla (FC 7); and for a fatty acyl CoA reductase (AAEL008125) in carcass (FC 138), gut (FC 6) and anal papilla (FC10), as shown in Additional file S2. These three enzymes were not detected in shed L4 cuticles (data in ProteomeXchange repository). Transcripts for several proteolytic enzymes were highly increased (FC > 100) notably in gut, but only one protein, a serine protease (AAEL001675) whose transcripts were increased in all three structures in BW L4 (Additional file S2), was detected by proteomics in both BW and FW shed L4 cuticles (data in ProteomeXchange repository). Transcripts for a metallo-endopeptidase was strongly decreased in gut (FC ≤ 0.01) and decreased in anal papilla and carcass (FC < 0.3), while those for a sterol desaturase were decreased in gut and carcass (FC < 0.1), and anal papilla (FC 0.4) in BW L4 (Additional file S2) with neither protein detected in shed L4 cuticles (data in ProteomeXchange repository).

### Ultrastructure of L4 and adult cuticles observed by TEM

**Fig. 1:**
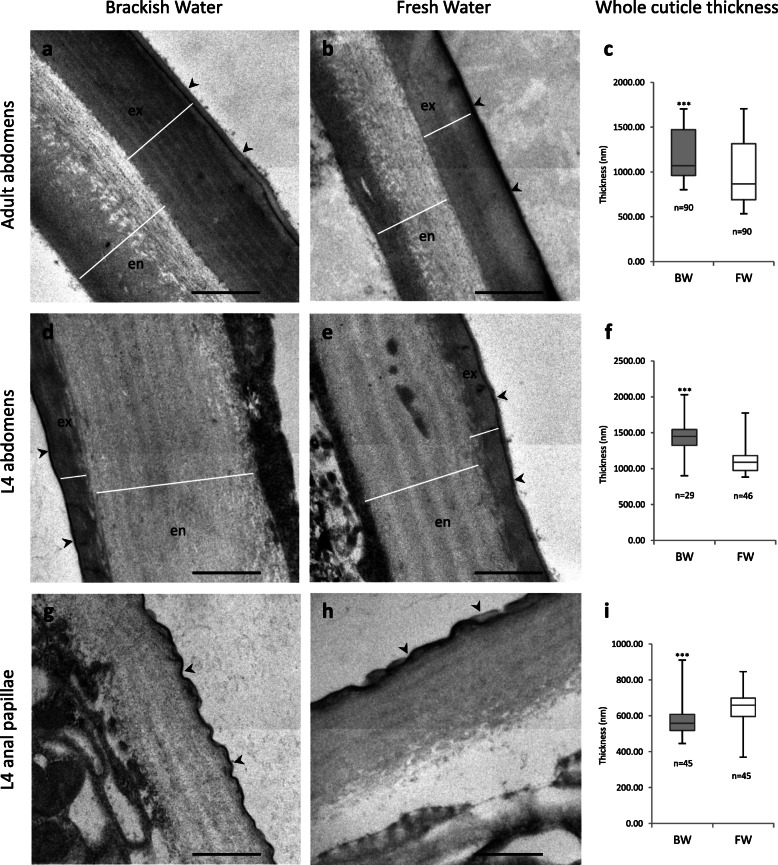
Cuticle ultrastructure by transmission electron microscopy. Legend Transmission electron micrographs of the cuticles in adult abdomen (**a**,**b**), L4 larval abdomen (**d**,**e**) and L4 anal papillae (**g**,**h**) from brackish (**a**,**d**,**g**) and fresh water (**b**,**e**,**h**) *Ae. aegypti.* Arrowheads mark the external surface. Box plots show the range (whiskers), median (horizontal line), and 25th and 75th percentile of measured thicknesses (box) of the whole cuticle of adult abdomen (**c**), L4 larval abdomen (**f**) and L4 anal papillae (**i**). n = total number of measurements (at least ten measurements per insect). *** *p*-value< 0.001 by the two-tailed Student’s t test. BW, brackish water; FW, fresh water; en, endocuticle; ex, exocuticle. Black scale bars represent 500 nm. White bars in **a**,**b**,**d **and **e** delineate the endocuticle and exocuticle

The cuticles of adult female and L4 6th abdominal sections, as well as the cuticle of L4 anal papillae of BW and FW *Ae. aegypti* specimens were observed by TEM (Fig. 1). Variations in whole cuticle thicknesses in different EM sections and between mosquito specimens within a rearing condition (BW or FW) constrained interpretation of the data on cuticle structural changes. The combined analysis of all measurements on adult abdomens (Fig. 1a-c) however suggested that (i) the whole cuticle was thicker (t = 6.3, *p* < 0.0001) in BW (1189 ± 58 nm, mean ± 95% confidence interval) than FW (973 ± 75 nm), and (ii) the endocuticle including its more electron lucent layer sometimes termed mesocuticle (t = 3.1, *p* = 0.0025; BW 648 ± 34 nm, FW 548 ± 55 nm), and the exocuticle (t = 6.1, p < 0.0001; BW 514 ± 29 nm, FW 424 ± 25 nm) were also thicker in BW adults. The cuticle also appeared thicker (t = 6.3, p < 0.0001; BW 1442 ± 86 nm, FW 1119 ± 58 nm) in BW L4 abdomens (Fig. 1d-f), but thinner (t = − 3.43, *p* = 0.0009; BW 577 ± 29 nm, FW 646 ± 29 nm) in BW L4 anal papillae (Fig. 1g-i). Considering all TEM sections, parallel sheets termed lamellae and helicoidally twisted sheets termed Bouligands that are formed from chitin microfibrils and chitin-binding cuticle proteins [45] tended to be more prominent in BW L4 than FW L4 cuticles.

## Discussion

The RNA-seq analysis identified many lncRNAs, some of which had markedly different expression levels in salinity-tolerant BW *Ae. aegypti* L4 compared to FW *Ae. aegypti* L4. Many other lncRNAs were identified with less prominent changes in FCs. Some lncRNAs showed noticeable variations in FCs between gut, anal papilla and carcass. As lncRNAs have important roles in regulating gene expression at the chromosome, transcription and post-transcription levels, further investigations into their functions in salinity tolerance in different *Ae. aegypti* larval tissues are warranted.

Receptors in mosquito larvae that sense environmental salinity have not been characterized. A notch homologue, a frizzled-type transmembrane receptor, a G-protein coupled receptor and a CREB regulatory factor, whose transcripts were strongly increased with FC ≥ 100 or decreased with FC ≤ 0.01 in BW L4 may have roles in sensing and adapting to salinity. Increases in transcripts for MAPK signaling pathway proteins, notably Jun and Jun kinase, and a POU-domain transcription factor in BW *Ae. aegypti* are consistent with observations on the short-term salinity response in anopheline L4 [44], and salinity responses in yeast [46] and brine shrimp [47]. Rho GTPases transduce extra-cellular signals to reorganize the cytoskeleton. Higher transcript levels for a Rho GTPase may therefore reflect a need for increased transport of vesicles containing cuticle components in BW. In addition, the differential expression of moulting-related protein hormones and their interacting proteins suggests that salinity-tolerance alters the complex interplay between ecdysone, JH, eclosion hormone and the ecdysis-triggering hormone in cuticle differentiation and moulting [48, 49]. Transcripts for several unannotated genes also showed marked FC changes (> 100 or ≤ 0.01) and the roles of their corresponding proteins in salinity tolerance merit further investigation. It is also evident that proteins derived from other transcripts with more modest FC changes may have functions in achieving salinity tolerance - a physiological state in BW *Ae. aegypti* that is likely to involve alterations in multiple biochemical pathways compared with FW *Ae. aegypti*.

Larval osmoregulation by anal papilla is facilitated by its thin cuticle, a syncytial epithelium and a lumen containing hemolymph. The transfer of FW *Ae. aegypti* L4 to 30% sea water increased hemolymph Na^+^, Cl^−^ and H^+^ and reduced Na^+^ and Cl^−^ uptake by anal papillae [34]. A V-type ATPase in the apical membrane that moves H^+^ out, a Cl^−^/HCO3^−^ exchanger that takes up Cl^−^, and a Na^+^/K^+^ ATPase located in the basal membrane of the anal papilla epithelium were identified as relevant transporters [34]. The expression of AQPs1–6 in the anal papilla was reported to be unaffected in *Ae. aegypti* exposed to BW [50]. We observed an increase in transcripts for AQP1, 3 and 4 as well as a putative AQP (AAEL021132) in anal papilla, gut and carcass in salinity-tolerant *Ae. aegypti* and the difference in the two observations requires further investigation. Detection of V-type ATPase and Na^+^/K^+^ ATPase subunits in both BW and FW cuticles in the proteomic analysis may be due to traces of epithelial membrane in shed cuticles. Transcripts for the α and β Na^+^/K^+^ ATPase subunits increased in all three structures which is consistent with greater active transport of ions in BW. Na^+^/H^+^ exchangers and NH_4_^+^ transporters present in anal papilla have been implicated in Na^+^, ammonia and H^+^ transport [51]. Higher levels of transcripts for an anion exchanger that also showed a greater anal papilla-specific increase in one transcript, and the Na^+^/H^+^ antiporter NHE3 in all three structures is consistent with findings for proteins with similar functions in whole L4 of *An. gambiae* in a short-term salinity response [44]. We find that transcript levels for NH_4_^+^ transporters were either unchanged or decreased, with the AeRh50.2 transporter transcript strongly decreased in all three structures in BW L4. In contrast, there was a prominent increase in the transcript for a cationic amino acid transporter in anal papilla with smaller increases in gut and carcass in BW L4. These findings suggest that ion transporters and AQPs in the different L4 structures function in the development of salinity-tolerance in *Ae. aegypti*.

Increased transcripts for allantoinase, a purine catabolizing enzyme, in all three L4 structures is consistent with findings from the short-term salinity response in anopheline L4 [44], and may reflect a greater catabolism of purines in salinity-tolerant *Ae. aegypti*.

A cuticle covers the external larval surface of larvae and the gut lumen excluding the midgut, and is also present in the tracheal lumen. The external cuticle is typically composed of (i) a 10-30 nm waxy water-proofing envelope on the outside, (ii) an underlying chitin-free epicuticle made up of highly cross-linked proteins, and (iii) a procuticle containing chitin microfibrils and cross-linked cuticle proteins [45], and composed of an exocuticle and endocuticle, generally synthesized just before and after ecdysis respectively [45, 52]. Epidermal cells assemble the cuticle and produce moulting fluid containing enzymes for separating the old cuticle from the newly formed one during ecdysis [45, 52]. The mRNA in mid-stage L4 code for proteins of the outer body wall endocuticle, tracheal cuticle and gut cuticle, and some pupal proteins including cuticle proteins in its exocuticle as well as most other L4 proteins. Protein expression in L4 is governed by the stability of mRNA, control of mRNA translation and protein half-life [53]. Proteins in the cuticles shed during the L4 to pupa metamorphosis will contain many exocuticle proteins made in L3 and endocuticle proteins synthesized in L4 [45, 52, 54]. However digestion by moulting fluid proteases leads to a relative loss of the endocuticle proteins in shed cuticles [39]. These factors lead to the observed lack of an exact correlation between the detection of individual cuticle proteins in shed L4 cuticles and their mid-L4 stage transcript levels.

Our results showed that the chitin-binding RR-1 and RR-2 family proteins were prominent among all the cuticle proteins and the 21 BW-specific cuticle proteins identified in shed L4 cuticles. The inability to detect FW cuticle-specific cuticle proteins, suggests that the 82 cuticle proteins that were identified as common to both BW and FW cuticles are normal L4 cuticle components of FW *Ae. aegypti.* The 23 proteins that are not identified as cuticle proteins and detected only in FW cuticles may either be present in BW cuticles below the threshold of detection or be down-regulated in BW. One candidate for downregulation is the epithelial NH_4_^+^ transporter AeRh50.2 which is detected only in FW cuticles and whose transcript is decreased in BW L4. However, the proteomics data show that salinity-tolerant *Ae. aegypti* are characterised by changes in protein composition, including those of cuticle proteins, in the L4 cuticle.

Cuticle proteins of *An. gambiae*, the best studied among mosquitoes, comprise > 298 proteins representing ~ 2% of all proteins coded in the *An. gambiae* genome [38, 39]. Many *An. gambiae* RR-1 and RR-2 genes are organized into co-expressed clusters in chromosomes [54]. Four clusters contained exclusively RR-1 genes were expressed within an instar, which is consistent with endocuticle synthesis at this time. Seven clusters which contained exclusively RR-2 genes showed peak expression immediately prior to ecdysis suggesting contribution to the exocuticle of the subsequent stage. Some RR-1 and RR-2 genes however had transcripts both immediately prior to and immediately after ecdysis and in different larval stages [54]. Besides chitin-binding, and possibly predominant localization within the endocuticle or exocuticle, defined functions have not yet been ascribed to an individual cuticle protein or cuticle protein family in mosquitoes [55–59]. The marked increase in transcripts for some cuticle proteins in all structures in BW L4 is consistent with observations in anopheline L4 subject to a short-term salinity stress [44]. The differences we observed in cuticle protein transcript FCs between gut, anal papilla and carcass probably reflect tissue-specialized responses to BW adaptation.

Both transcriptome and proteome analyses suggest that changes in RR-2 expression are important for salinity tolerance in *Ae. aegypti* L4, particularly in the external surface cuticles present in the carcass and anal papilla. We hypothesize that an increase in specific members of the RR-2 family reflects a key role for these proteins in remodeling the larval procuticle, which is also supported by the TEM observations, to reduce its permeability to water and ions in salinity-tolerant *Ae. aegypti*. The sharp peak in expression of some RR-2 mRNAs in late stages of L4 in *An. gambiae* [54] is pertinent because their earlier expression in mid-L4 BW *Ae. aegypti* may help confer the greater cuticle impermeability that is characteristic of pupae [29] to L4. We separately discuss below the likely accompanying changes in the envelope and epicuticle that can also reduce cuticle permeability in BW L4. Such changes in L4 may conceivably then be carried through to pupal and adult cuticles.

Golgins participate in transporting secretory vesicles from the endothelial Golgi to the plasma membrane [60] and Osiris proteins in cuticle formation [42]. The observed rise in the mRNA levels for both types of proteins in BW L4 is consistent with increased synthesis of cuticle components. Chitin is a major constituent synthesized during the formation of the procuticle and degraded during ecdysis. The increase in chitin synthase transcripts in all three structures in BW L4 is consistent with enhanced cuticle synthesis, which may also be related to the marked increase in a chitin-binding protein transcript in gut and smaller increases in anal papilla and carcass. Chitinase transcripts were however increased only in anal papilla suggesting that chitin metabolism may be different in BW L4 anal papilla. This and other anal papilla-specific molecular changes observed in our study may be related to the enlargement of anal papillae in BW *Ae. aegypti* [17], specific alterations in anal papilla ion and water transport in BW [34], and a possible thinner cuticle in anal papilla of BW L4 observed here by TEM, and merit further investigation. Proteomics of shed *Ae. aegypti* L4 cuticles identified many OPACs corresponding to proteins shown to be present in *An. gambiae* cuticles [38, 39]. OPACs that showed markedly altered transcript levels in BW L4 stage may contribute to cuticle structural changes in BW *Ae. aegypti.* Such OPACs included enzymes for melanization and sclerotization, muscle and cytoskeletal proteins, C-type lectins, potential moulting fluid proteases, chitinase, and glucose dehydrogenase as well as the cuticle structural proteins cuticulin and resilin.

Marine mosquitoes *Opifex fuscus* and *Aedes detritus* that normally develop in saline water have more water-impermeable body wall cuticles than FW arthropods [61, 62]. Greater impermeability in the body wall cuticle of salinity-tolerant *Ae. aegypti* larvae in comparison to FW *Ae. aegypti* has yet to be experimentally demonstrated. Our findings suggest that further investigations on structural and functional changes in cuticles lining the gut, trachea and AP, in addition to the body wall cuticle, are important for understanding salinity tolerance in *Ae. aegypti*.

The epicuticle and its waxy envelope, containing respectively tanned cuticulins and both straight chain and methyl-branched long chain hydrocarbons, make a large contribution to water impermeability in arthropod cuticles [63, 64]. Long chain hydrocarbons are produced in *An. gambiae* by elongation of fatty acids followed by reduction reactions involving cytochrome P450 of the CYP4G family [64, 65]. Increased synthesis of long chain hydrocarbons in BW L4 is supported by the large increases observed in transcripts for fatty acid synthase, very long chain fatty acid elongase, fatty acid acyl CoA reductase and the CYP4G17 homolog. Together with the marked increase in cuticulin transcripts, these transcriptomic findings suggest that augmentation of the water proofing epicuticle and its waxy envelope in the body wall, and possibly also the tracheal system, is important for salinity tolerance in *Ae. aegypti* larvae. Activation of the MAPK signaling pathway in BW L4 is consistent with the pathway’s role in activating oenocytes to synthesize epicuticular lipid components [66]. Changes in the composition of cuticulins in the epicuticle, lipids in the waxy envelope, cuticle proteins (notably of RR-2s) in the procuticle, OPACs and chitin suggested by the transcriptomic and proteomic findings indicate that the cuticle structure is altered in BW L4. The TEM observations are also consistent with changes in the structure of external procuticles in BW L4, including their lamellae and Bouligands that are formed from chitin microfibrils and chitin-binding cuticle proteins such as RR-2s [40, 45]. The marked changes in the levels of many other transcripts in BW L4 may make both cuticle-related and cuticle-independent contributions to salinity tolerance in *Ae. aegypti* L4. All these changes can contribute to the higher LC_50_ for salinity shown by BW *Ae. aegypti* larvae [9, 16]. Because of the heritability of larval salinity tolerance in *Ae. aegypti* [16], further investigations on the genomics of salinity-tolerance in *Ae. aegypti* are warranted.

Cuticle thickening has been associated with insecticide resistance in mosquitoes. Pyrethroid – resistant strains of adult *An. funestus* and *An. gambiae* have thicker external cuticles and reduced cuticular penetration of pyrethroids than sensitive strains [67–69]. Similar observations were made on larvae of the oriental fruit fly *Bactrocerca dorsalis* [70]. Cuticle protein changes have been suggested to contribute to thicker cuticles in adult pyrethroid-resistant *An. gambiae* [68, 69]. The procuticle thickening and other cuticle changes that seems to occur in BW L4 and adult female *Ae. aegypti*, can potentially result in greater resistance to larval and adult insecticides. Larvae of salinity-tolerant *Ae. aegypti* [11] and *An. aquasalis* [71] also show reduced sensitivity to the midgut-acting *Bacillus thuringiensis* endotoxin, a commonly-used larvicide. A cuticle that reduces water and ion permeability in salinity-tolerant larvae may also reduce absorption of the organophosphate Temephos, the most widely-used larvicide for larval source reduction of FW *Ae. aegypti* worldwide. Reduced susceptibility to common larvicides combined with the neglect of BW habitats for larval source reduction, can lead to the spread of salinity-tolerant *Ae. aegypti* populations in coastal areas and an increase in the transmission of arboviral diseases. Rising sea levels that expand coastal BW habitats [19–23] will exacerbate this process. Further studies of cuticle ultrastructure and insecticide resistance in preimaginal stages and adults of salinity-tolerant *Ae. aegypti* are therefore needed in this context.

The findings in salinity-tolerant *Ae. aegypti* may also apply to the salinity-tolerant *Ae. albopictus* and anophelines recently detected in the Jaffna peninsula [9, 11, 16]. Similar BW-adaptive changes to those in *Ae. aegypti* occurring in FW anophelines accompanied by reproductive isolation in coastal areas may have been the origin of salinity-tolerant species like *An. merus* in Africa [72, 73], *An. sundaicus* in Asia [74] and *An. aquasalis* in America [19, 20]. However, salinity tolerance in *Ae. aegypti* which involves heritable changes [16] has not yet prevented interbreeding and gene flow with FW *Ae. aegypti* in the rapidly salinizing Jaffna peninsula [16]. The spread of the salinity-tolerant trait in the peninsula is shown by *Ae. aegypti* collected in FW ovitraps in the peninsula demonstrating a higher LC_50_ for salinity than those collected from mainland Sri Lanka [9]. Salinity-tolerant *Ae. aegypti* originating in the Jaffna peninsula can also readily expand their range to coastal areas of mainland Sri Lanka in the future [23].

## Conclusions

Salinity-tolerance in *Ae. aegypti* is characterized by differences in the comparative transcriptomics profiles of gut, anal papilla and carcass, notably for cuticle and cuticle-associated proteins, as well as signalling pathway proteins and other effector molecules. RNA-seq analysis on large pools of mosquito structures under two different biological conditions has yielded important information in other comparative transcriptomic studies [75, 76] and is cost effective [77]. However, the use of biological replicates and/or RT-qPCR can better demonstrate changes in the expression of specific transcripts and their statistical significance. Salinity tolerant *Ae. aegypti* also showed differences in larval cuticle proteins composition by proteomics and larval and adult cuticle ultrastructure by transmission electron microscopy that were compatible with the transcriptomic results. The findings show the need for additional investigations on cuticle structure and function in relation to insecticide resistance and the genomic biology of salinity tolerance in *Ae. aegypti*. The observations in the principal global arboviral vector *Ae. aegypti* have fundamental biological and multiple epidemiological implications in the context of rising sea levels caused by climate change expanding coastal brackish water habitats. There are attendant consequences also for other FW mosquito vectors and the diseases they transmit.

## Methods

### *Aedes aegypti* for experiments

Self-mating BW and FW laboratory colonies of *Ae. aegypti* were established with larvae collected from BW and FW habitats in the Jaffna peninsula of Sri Lanka [16], respectively. For oviposition, egg hatching and preimaginal development into adults, FW and BW *Ae. aegypti* were maintained in tap water and sea water diluted to 10 ppt salt with tap water, respectively [16]. During the present experiments, the L1, L2, L3, L4 and pupal stages lasted approximately 48 h, 48 h, 72 h, 72 h and 24 h in FW *Ae. aegypti*, respectively*.* BW *Ae. aegypti* differed only in having more prolonged L2 and pupal stages of approximately 72 h and 24-36 h, respectively.

### Transcriptomics of L4 larvae

Individual L4, 36-40 h after ecdysis, from the 31st-FW and 28th-BW generations after colony establishment were dissected to yield (i) whole gut including associated Malpighian tubules (gut); (ii) four anal papillae; and (iii) rest of the carcass which contains most of the trachea (carcass). These were placed directly into RNAlater® solution (Ambion, Austin, TX). RNA was extracted separately from pools of 35–40 of each of the three mosquito structures from FW and BW larvae using the HiPurATM Total RNA Miniprep kit (Himedia, Mumbai, India). Pooling a large number of mosquitoes mitigates the need for biological replicates of libraries in comparative transcriptome profiling of specific mosquito structures in two biological conditions as described for *An. gambiae* [75] and *Ae. aegypti* [76]. Such pooling can retain statistical power while minimizing the cost of RNA-seq experiments [77]. Extracted RNA was sent in RNAstable® tubes (Biomatrica, CA, USA) to Macrogen (Seoul, South Korea) for cDNA library preparation and DNA sequencing. Illumina cDNA libraries were prepared using TruSeq RNA from poly(A)-selected RNA, and sequencing performed using Illumina Hiseq with 100 bp read lengths and sequence depths of > 40 million reads per sample. Before mapping raw reads were subjected to removal of the adaptor sequences using Trim Galore tool, and further reads were filtered using a sliding window for average quality of 20 within the window of 4 bases and reads below of 90 bp were dropped out using Trimmomatic flexible read trimming tool. Paired end reads were mapped to the *Ae. aegypti* Liverpool AGWG strain transcripts AaegL5.1 in VectorBase (www.vectorbase.org) with the Galaxy Interface bowtie tool (www.usegalaxy.org) using default parameters allowing up to two mismatches per 28 bp seed (Galaxy version 1.1.2). Summary of mapping statistics are provided in Additional file S5. Transcript abundance were extracted as read counts using SAMTools pileup [78]. Reads per million mapped reads (rpm) and the ratio of rpms in BW to FW termed fold change (FC) were calculated for every transcript. Cuticle protein annotation was according to VectorBase or manually done where necessary with the CutProtFam-Pred tool (http://aias.biol.uoa.gr/CutProtFam-Pred/home.php) [79, 80].

### Proteomics of shed L4 cuticles

Cuticles cast from L4 when they transformed into pupae in the 41st-BW and 43rd-FW generations after colony establishment were collected, rinsed five times in distilled water and transferred to cryo-vials (~ 45 cuticles per vial). Cuticles were collected in triplicate and stored at − 80 °C before freeze drying for couriering to Platform BioPark Archamps.

For proteomics analysis, the following reagents were used: RapiGest SF surfactant (Waters, Milford, MA), reagent grade NH_4_HCO_3_, hexofluoroisopopanol (HFIP), 4-vinylpyridine (4-VP), dithiothreitol (DTT), LCMS-grade formic acid (FA) from Sigma-Aldrich (St. Louis, MO), MilliQ water (Merck Millipore, Billerica, MA), acetonitrile (ACN) and trifluoroacetic acid (TFA) of HPLC grade or higher from Carlo Erba Reagents (Val de Reuil, France), PBS buffer from Thermo Fisher Scientific (Waltham, MA), and sequencing grade modified trypsin (Promega, Madison, WI).

Proteins were extracted from mosquito cuticles following an established protocol [81]. Briefly, dried samples were incubated in HFIP for 4 h at 4 °C. HFIP was evaporated, and samples were incubated overnight at 4 °C in 50 mM NH_4_HCO_3_ (pH 7.8) supplemented with 0.1% RapiGest SF. Proteins were reduced with 30 mM DTT in 50 mM NH_4_HCO_3_ for 1 h in the dark at 56 °C prior to alkylation with 95 mM 4-VP for 1 h in the dark at room temperature. Digestion was carried out overnight at 37 °C with 0.5 μg of trypsin. To stop proteolysis and cleave RapiGest SF, samples were transferred into clean 1.5 mL LoBind tubes (Eppendorf), acidified with TFA and incubated for 30 min at 37 °C. Finally, samples were dried under CentriVap vacuum (Labconco, Kansas City, MO) and the dried pellets resuspended in 2%ACN/0.1% TFA. NanoLC-MS/MS analysis was then carried out as described [81] in an Ultimate 3000 nano-HPLC, coupled with a Q-Exactive Orbitrap high resolution mass spectrometer (unless stated otherwise, all hardware, software and consumables were from Thermo Fisher Scientific, MA). Samples were loaded onto a C_18_ PepMap100 precolumn (5 μm, 300 μm × 5 mm) at 10 μL min^− 1^ and separated in an Acclaim C_18_ PepMap100 column (3 μm, 75 μm × 250 mm) at a flow rate of 300 nL min^− 1^. Peptides were eluted in a biphasic linear gradient of water/ACN/0.1% FA (v/v), with 2–32% and of 32–65% ACN (0.1% FA) in 100 and 5 min, respectively. The Q-Exactive mass spectrometer, equipped with a nanospray ion source, was used in positive mode and data-dependent acquisition. The voltage applied to the nano-tips was adjusted to produce 0.3 μA and the entrance capillary was maintained at 300 °C. The Q-Exactive Orbitrap acquired a full-range scan from 380 to 2000 *m/z* (70,000 resolution, automatic gain control (AGC) target 3 × 10^6^, maximum ion trap time (IT) 200 ms) and then fragmented the top ten-peptide ions in each cycle (17,500 resolution, AGC target 2 × 10^5^, maximum IT 100 ms, intensity threshold 4 × 10^4^, excluding charge-unassigned ions, Normalized Collision Energy of 30). Parent ions were excluded from MS/MS for the next 15 s. The software Chromeleon Xpress and Xcalibur 2.2 were used to control the HPLC and the mass spectrometer, respectively. One-tenth of each digested sample was injected for LC-MS/MS analysis, and three technical replicates were acquired with each sample.

Sequest HT was run by Proteome Discoverer 2.4 (Thermo Fisher Scientific) to match the acquired MS/MS spectra to a protein database of the full mosquito taxon available from Uniprot, downloaded on 1 April 2019 (UniprotKB + TrEMBL, total 237,216 entries). The following parameters were used: trypsin digest with two maximum missed cleavages; six and 144 amino acids as minimum and maximum peptide lengths, respectively; a tolerance of 10 ppm/0.02 Da for precursors and fragment ions, respectively; cysteine pyridyl-ethylation was set as a fixed modification; C-terminal protein amidation, methionine and tryptophan oxidation were set as variable modifications. The identification confidence was set at a false discovery rate of 1%. Proteins consistently identified across a series of three technical replicates were considered correctly identified. Cuticle proteins identified from mosquitoes other than *Ae. aegypti* were used in BLASTp analysis online at NCBI (https://blast.ncbi.nlm.nih.gov/Blast.cgi?PAGE=Proteins) and then VectorBase to identify homologous *Ae. aegypti* proteins and genes. Protein sequences were submitted online to CutProtFam-Pred tool [79, 80] to retrieve predicted cuticle proteins.

### Transmission electron microscopy of cuticles

L4 and adult females were collected 5-10 h and 8-10 h post-ecdysis from the 53rd-FW and 54th-BW generations, respectively. Intact 6th-abdominal segment from each and anal papillae from the L4 were dissected, fixed in 0.1 M sodium cacodylate (Sigma-Aldrich, MO, USA) buffer pH 7.2 (FB) containing 4% glutaraldehyde (Sigma-Aldrich, MO, USA) for 4 h at 4 °C, rinsed three times in FB, and then stored at 4 °C in the same buffer containing 0.5% glutaraldehyde. Samples were rinsed with FB, post-fixed in FB containing 1% osmic acid for 2 h at 4 °C, included in 3% low melting agarose, and further dehydrated in a graded series of ethanol solutions (30–100%). Finally, samples were embedded in EmBed 812 using an automated microwave tissue processor for electron microscopy, Leica EM AMW. Sections 65 nm thick were observed in a JEOL JEM1400 microscope. Three samples each from BW and FW specimens were observed. Measurements of cuticle layers from EM sections were done using Fiji [82], analyzed with at least 10 measurements per sample, and three samples for adult abdomens and L4 anal papillae. Fewer measurements were made on L4 abdomen as the thickness was only measured when the cuticle was in direct contact with epidermal cells (one sample, 29 measurements for BW; and two samples, 46 measurements for FW). The significance of differences in cuticle layer thicknesses was determined by the two-tailed Student’s t test for independent samples.

## Supplementary Information

**Additional file 1. **Complete RNA-seq analysis. This table shows all 30,485 transcripts identified in gut, anal papilla and carcass, in BW and FW *Ae. aegypti* arranged in descending order of FC in carcass.

**Additional file 2. **Transcripts with FC > 100 or ≤ 0.01 in RNA-seq analysis. This table shows all the transcripts with FC > 100 or ≤ 0.01 in gut, anal papilla and carcass in BW and FW *Ae. aegypti* arranged in descending order of FC in each structure.

**Additional file 3.** Cuticle proteins in shed L4 cuticles. This table shows A. numbers of proteins identified in shed BW and FW L4 cuticles; B. cuticle protein families identified in shed L4 cuticles; C. details of the different cuticle proteins identified in shed BW L4 cuticles with their corresponding transcriptomic data, i.e. rpm and FCs in gut, anal papilla and carcass; D. gene identity of all cuticle proteins identified in BW and FW shed cuticles by proteomics.

**Additional file 4. **RNA-seq analysis of specific non-cuticle proteins. This table summarizes transcriptomic and proteomic data for specific non-cuticle proteins that may have a role in salinity adaptation in *Ae. aegypti* L4, excluding those whose transcripts have large FC changes which are shown in Additional file S2.

**Additional file 5.** Mapping data for the RNA-seq libraries. This document tabulates the relevant mapping data for the six RNA-seq libraries used for transcriptomic analyses.

## Data Availability

The datasets supporting the conclusions of this article are available as follows (i) Illumina sequencing data as BioProject PRJNA629452 with BioSample accession SAMN14771163 and SRA accessions SRR11661571 to SRR11661576 for the six libraries at NCBI (https://www.ncbi.nlm.nih.gov/bioproject), and (ii) the mass spectrometry proteomics data at ProteomeXchange Consortium (http://www.proteomexchange.org) via PRIDE partner repository under accession PXD018397 and Project doi:10.6019/PXD018397. Other data supporting the conclusions of this article are either included within the article or provided in Additional files 1, 2, 3, 4 and 5.

## Abbreviations

AQP: Aquaporin
BW: Brackish water;
FC: Fold change
FW: Fresh water
JH: Juvenile hormone
L4: Fourth instar larva
lncRNA: Long non-coding RNA
MAPK: Mitogen-activated protein kinase
rpm: Reads per million mapped reads
OPAC: Other protein associated with cuticle
RT-qPCR: Reverse transcription quantitative polymerase chain reaction
TEM: Transmission electron microscopy
