# Supplementary material for: Transcriptomic, proteomic and ultrastructural studies on salinity-tolerant Aedes aegypti in the context of rising sea levels and arboviral disease epidemiology
Source: BMC Genomics. 2021 Apr 9;22:253. doi: 10.1186/s12864-021-07564-8 (PMC8034070; doi:10.1186/s12864-021-07564-8)
Supplement: Supplementary file 5 — Additional file 5. Mapping data for the RNA-seq libraries. This document tabulates the relevant mapping data for the six RNA-seq libraries used for transcriptomic analyses. [file 12864_2021_7564_MOESM5_ESM.docx]

ADDITIONAL FILE S5 - Mapping Data for the RNA-Seq libraries

Anal Papilla FW

48993336 + 0 in total (QC-passed reads + QC-failed reads)

0 + 0 secondary

0 + 0 supplementary

0 + 0 duplicates

31589086 + 0 mapped (64.48%:-nan%)

48993336 + 0 paired in sequencing

24496668 + 0 read1

24496668 + 0 read2

31589086 + 0 properly paired (64.48%:-nan%)

31589086 + 0 with itself and mate mapped

0 + 0 singletons (0.00%:-nan%)

0 + 0 with mate mapped to a different chr

0 + 0 with mate mapped to a different chr (mapQ>=5)

Anal Papilla BW

57453146 + 0 in total (QC-passed reads + QC-failed reads)

0 + 0 secondary

0 + 0 supplementary

0 + 0 duplicates

34901658 + 0 mapped (60.75%:-nan%)

57453146 + 0 paired in sequencing

28726573 + 0 read1

28726573 + 0 read2

34901658 + 0 properly paired (60.75%:-nan%)

34901658 + 0 with itself and mate mapped

0 + 0 singletons (0.00%:-nan%)

0 + 0 with mate mapped to a different chr

0 + 0 with mate mapped to a different chr (mapQ>=5)

Carcass FW

48515842 + 0 in total (QC-passed reads + QC-failed reads)

0 + 0 secondary

0 + 0 supplementary

0 + 0 duplicates

33120798 + 0 mapped (68.27%:-nan%)

48515842 + 0 paired in sequencing

24257921 + 0 read1

24257921 + 0 read2

33120798 + 0 properly paired (68.27%:-nan%)

33120798 + 0 with itself and mate mapped

0 + 0 singletons (0.00%:-nan%)

0 + 0 with mate mapped to a different chr

0 + 0 with mate mapped to a different chr (mapQ>=5)

Carcass BW

53801418 + 0 in total (QC-passed reads + QC-failed reads)

0 + 0 secondary

0 + 0 supplementary

0 + 0 duplicates

35509936 + 0 mapped (66.00%:-nan%)

53801418 + 0 paired in sequencing

26900709 + 0 read1

26900709 + 0 read2

35509936 + 0 properly paired (66.00%:-nan%)

35509936 + 0 with itself and mate mapped

0 + 0 singletons (0.00%:-nan%)

0 + 0 with mate mapped to a different chr

0 + 0 with mate mapped to a different chr (mapQ>=5)

Gut FW

46431604 + 0 in total (QC-passed reads + QC-failed reads)

0 + 0 secondary

0 + 0 supplementary

0 + 0 duplicates

31722610 + 0 mapped (68.32%:-nan%)

46431604 + 0 paired in sequencing

23215802 + 0 read1

23215802 + 0 read2

31722610 + 0 properly paired (68.32%:-nan%)

31722610 + 0 with itself and mate mapped

0 + 0 singletons (0.00%:-nan%)

0 + 0 with mate mapped to a different chr

0 + 0 with mate mapped to a different chr (mapQ>=5)

Gut BW

47587650 + 0 in total (QC-passed reads + QC-failed reads)

0 + 0 secondary

0 + 0 supplementary

0 + 0 duplicates

32194726 + 0 mapped (67.65%:-nan%)

47587650 + 0 paired in sequencing

23793825 + 0 read1BW

32194726 + 0 with itself and mate mapped

0 + 0 singletons (0.00%:-nan%)

0 + 0 with mate mapped to a different chr

0 + 0 with mate mapped to a different chr (mapQ>=5)

Summary Table

|  | Anal Papilla FW | AP Papilla BW | Gut FW | Gut BW | Carcass FW | Carcass BW |
| --- | --- | --- | --- | --- | --- | --- |
| Total | 48993336 | 57453146 | 46431604 | 47587650 | 48515842 | 53801418 |
| Paired | 31589086 | 34901658 | 31722610 | 32194726 | 33120798 | 35509936 |
| Percentage | 64.48% | 60.75% | 68.32% | 67.65% | 68.27% | 66.00% |
